# The Relationship of Photopic and Mesopic Contrast Sensitivity to Retinal–Choroidal Structural Characteristics in Low-to-Moderate and High Myopia

**DOI:** 10.1167/tvst.14.12.6

**Published:** 2025-12-02

**Authors:** Meng Lin, Yadong Huang, Jian Zhao, Minzhi Xiao, Hui Wang, Charong He, Kechun Liu, Fan Lu, Liang Hu

**Affiliations:** 1 National Clinical Research Center for Ocular Diseases, Eye Hospital, Wenzhou Medical University, Wenzhou, People's Republic of China; 2 National Engineering Research Center of Ophthalmology and Optometry, Eye Hospital, Wenzhou Medical University, Wenzhou, People's Republic of China

**Keywords:** myopia, contrast sensitivity (CS), mesopic vision, choroidal vascularity, optical coherence tomography (OCT)

## Abstract

**Purpose:**

This study aimed to compare photopic and mesopic contrast sensitivity (CS) between low-to-moderate myopia (LMM) and high myopia (HM) groups, and to evaluate the associations between CS and chorioretinal structural and vascular parameters.

**Methods:**

In this cross-sectional study, 108 participants were divided into an LMM group (*n* = 53, mean age = 21.0 years, mean SE = −4.25 diopters [D]) and an HM group (*n* = 55, mean age = 24.0 years, mean SE = −8.25 D). CS was tested under photopic (85 cd/m²) and mesopic (3 cd/m²) conditions. Chorioretinal parameters were measured using swept-source optical coherence tomography/optical coherence tomography angiography (SS-OCT/OCTA). Associations were assessed using univariate and stepwise multiple linear regression analyses.

**Results:**

The HM group exhibited significantly reduced CS under both photopic (1.07 ± 0.11 vs. 1.19 ± 0.09 in the LMM group, *P* < 0.001) and mesopic (median = 0.64 vs. 1.04 in the LMM group, *P* < 0.001) conditions. Compared to the LMM group, the HM group had a significantly thinner choroid and a higher choroidal vascularity index (CVI). Mesopic CS (CSm) demonstrated stronger and more extensive associations with chorioretinal parameters than photopic CS (CSp).

**Conclusions:**

Chorioretinal alterations in high myopia appear to have a more profound impact on visual function under mesopic than photopic conditions. Given its sensitivity to both choroidal and retinal alterations, mesopic CS is a parameter that warrants further investigation for its potential in assessing functional impairment in myopia.

**Translational Relevance:**

Measuring mesopic contrast sensitivity offers a superior functional indicator of underlying chorioretinal health in myopia, allowing for earlier and more comprehensive assessment of visual decline in clinical practice.

## Introduction

Myopia and high myopia represent widespread refractive anomalies, with their prevalence steadily increasing in tandem with the acceleration of globalization and shifts in lifestyle patterns.^1^^,^^2^ This escalating trend not only compromises individual visual health by heightening the risk of ocular pathologies and blindness but also imposes significant socioeconomic burdens on society.^3^^,^^4^

As the severity of myopia intensifies, the elongation of axial length (AL) precipitates a cascade of structural modifications within the retinal and choroidal regions of the fundus. These structural alterations encompass variations in retinal thickness, remodeling of choroidal vasculature, and adjustments in hemodynamic parameters. Such modifications not only signify the progression of myopia but also possess the potential to directly impair visual function.^5^^,^^6^ Specifically, retinal thinning, alterations in choroidal vascular density, and heterogeneous blood perfusion may result in inadequate metabolic support for retinal neurons, subsequently compromising the processing and transmission of visual signals. Ultimately, these changes culminate in a diminution of visual function.^7^^–^^9^ Consequently, the precise assessment of visual function in myopic eyes, particularly metrics that can reflect early or subtle changes, is of significant clinical importance.

The Contrast Sensitivity Function (CSF) constitutes an essential metric for evaluating the visual system’s ability to process varying luminance levels and spatial frequencies, thereby playing a pivotal role in myopia research.^6^ It is well-established that myopia impairs contrast sensitivity (CS) under standard photopic conditions.^10^^,^^11^ However, existing studies have predominantly focused on photopic conditions, leading to a relative neglect of mesopic visual function.^12^^–^^14^ Whereas some studies have noted that CS declines as lighting conditions change,^15^ the fundamental link between this functional impairment under mesopic light and the specific microstructural alterations in the myopic retina and choroid remains largely unknown.

Mesopic lighting, with illumination intermediate between photopic and scotopic environments, relies on the synergistic function of both rod and cone photoreceptors.^16^ This characteristic may render mesopic CS changes more complex and potentially more revealing of early visual deficits inconspicuous under photopic conditions. Literature suggests that under certain pathological states involving retinal metabolic stress or structural compromise, mesopic functions can exhibit heightened sensitivity.^17^ Given that myopia is often associated with choroidal thinning and potential underperfusion, this could challenge the highly metabolic photoreceptors, particularly rod cells active under mesopic conditions. Therefore, investigating alterations in mesopic CSF in individuals with myopia is critical for comprehensively understanding myopia’s visual impact and for identifying more sensitive visual function indicators.

Therefore, the primary objectives of this study are twofold: first, to delineate the differences in CS function between individuals with high myopia (HM) and low-to-moderate myopia (LMM) under photopic and mesopic lighting conditions; and second, to examine the correlations between alterations in CS under these conditions and the microstructural parameters of the retina and choroid. This investigation seeks to explore the associations between retinal-choroidal structural and vascular parameters and visual function under different lighting conditions in myopia, which may provide insights for future mechanistic studies and inform clinical recommendations for the management and monitoring of patients with myopia.

## Materials and Methods

### Patients

This cross-sectional study was approved by the Ethics Committee of the Eye Hospital affiliated with Wenzhou Medical University (Approval No. 2023-233-K-183-01). From November 2021 to July 2023, a total of 108 participants were enrolled from the optometry clinic population of the Eye Hospital. All procedures adhered to the principles outlined in the Declaration of Helsinki. Written informed consent was obtained from each participant, and for those under 18 years of age, from their parents or legal guardians.

Prior to study enrollment, all participants underwent comprehensive ophthalmic evaluations, including non-cycloplegic subjective refraction, anterior segment examinations, detailed ocular health assessments, AL measurements, and intraocular pressure (IOP) determinations. Pupil size was measured under standardized scotopic conditions using an iTrace aberrometer (Tracy Technologies, USA). Exclusion criteria encompassed an IOP exceeding 21 millimeters of mercury (mm Hg), best-corrected visual acuity (BCVA) lower than 20/20, the presence of clinically significant lens opacity or other media opacities, presence of fundus retinal pathologies, glaucoma, any history of intraocular surgery, severe high-myopia–related complications (e.g. myopic maculopathy, retinoschisis, and choroidal neovascularization), relevant systemic comorbidities, or ongoing myopia control interventions such as orthokeratology or atropine therapy. To ensure standardized conditions for choroidal imaging, participants were instructed to refrain from consuming caffeine and alcohol for 24 hours prior to their examination.

Eligible participants were then assigned to one of two groups: (1) the LMM group, defined as a myopic prescription within −6.00 diopters (D), and (2) the HM group, defined as a spherical equivalent (SE) of −6.00 D or greater, without concomitant myopic macular lesions.

### Contrast Sensitivity Measurement

The CS was evaluated using the CSV-1000 test chart (Vector Vision, USA),^18^^,^^19^ which presents sine-wave gratings at 4 spatial frequencies: 3, 6, 12, and 18 cycles per degree (cpd). Prior to both photopic and mesopic testing, optimal refractive correction was achieved for each participant using trial lenses to attain BCVA. For photopic assessment, participants underwent a 5-minute light adaptation period at an ambient room illumination of 500 lux, followed by photopic contrast sensitivity testing performed with the CSV-1000 chart set to a standardized background luminance of 85 cd/m². For mesopic assessment, participants underwent a 5-minute dark adaptation period in complete darkness (<1 cd/m²), followed by mesopic CS testing conducted at a background luminance of 3 cd/m². All tests were performed monocularly (right eye) at a viewing distance of 2.5 meters. All CS measurements, both photopic and mesopic, were performed without glare.

For each participant, three consecutive measurements were obtained from the right eye and then averaged. From these results, the CSF was plotted, and the area under the log contrast sensitivity function (AULCSF) was calculated to provide a quantitative index of the overall CS performance. The AULCSF value obtained under photopic conditions was designated as AULCSFp (CSp), whereas the AULCSF value measured under mesopic conditions was designated as AULCSFm (CSm).

### Swept-Source Optical Coherence Tomography and Optical Coherence Tomography Angiography Measurements

An optical coherence tomography/ swept-source optical coherence tomography angiography (OCT/SS-OCTA) system (VG100D, SVision Imaging, Henan, China) was used to quantify macular structural and vascular parameters.^8^^,^^20^ This apparatus incorporates a swept-source light with a central wavelength of 1050 nm, a scanning rate of 200,000 A-scans per second, an axial resolution of 5 µm, a transverse resolution of 13 µm, and a tissue penetration depth of up to 6 mm.

Utilizing a raster scanning protocol, the thicknesses of various macular layers were ascertained, including total retinal thickness (RET), nerve fiber layer thickness (RNFLT), ganglion cell complex thickness (GCIPLT), inner nuclear layer thickness (INLT), outer retinal thickness (ORT), and choroidal thickness (ChT). Additionally, parameters such as inner retinal flow (IRF), choriocapillaris flow (CCF), choroidal vascular index (CVI), and choroidal vascular volume (CVV) were quantified. The imaging region encompassed a 6 mm × 6 mm zone centered on the fovea, with a scanning depth of 6 mm. Following the Early Treatment Diabetic Retinopathy Study (ETDRS) grid methodology, the macular region was partitioned into 3 concentric zones—1, 3, and 6 mm in diameter—corresponding, respectively, to the foveal, parafoveal, and perifoveal areas.

Retinal and choroidal layer thicknesses were segmented and measured using the system's integrated software. All automated segmentations, particularly for the choroidoscleral interface and inner or outer retinal boundaries, were independently reviewed by two trained graders. Manual corrections were performed according to predefined criteria when significant deviations in automated segmentation were identified. Scans with a signal strength index below 8 or those containing significant motion, blink, or projection artifacts that could not be effectively removed by the built-in motion correction algorithms were excluded from the analysis. For vascular parameters, the choriocapillaris perfusion area was delineated as the region extending from 10 µm above Bruch's membrane to 25 µm below it, whereas the retinal inner-layer perfusion area was defined as the region from 5 µm above the internal limiting membrane to 25 µm below the inner nuclear layer.^21^^,^^22^ Using the system's embedded algorithms, CVV was isolated, and CVI—defined as the ratio of CVV to the total choroidal volume—was subsequently calculated.

To standardize imaging metrics and mitigate the influence of AL on measurement accuracy, the Bennett formula (t = p × q × s) was utilized, where t is the actual scan length, p represents the OCT system's magnification factor, q denotes the eye-specific magnification factor, and s corresponds to the system's original measurement values. This calibration procedure ensured enhanced reliability and precision in all quantitative assessments.

### Statistical Analysis

All statistical computations were performed using SPSS Statistics 27.0 (IBM, Armonk, NY, USA). A priori sample size estimation indicated that a minimum of 17 subjects per group was required to achieve 90% statistical power at a 2-sided α level of 0.05. The final cohort of 108 participants substantially exceeded this minimum requirement, ensuring robust statistical power for the analyses. The Kolmogorov–Smirnov test was used to evaluate data normality. Variables conforming to normal distributions were presented as means ± standard deviations, whereas those demonstrating non-normal distributions were summarized as medians and interquartile ranges (IQRs). Categorical variables were reported as frequencies and proportions.

For correlation analyses, univariate linear regression analyses were first performed to examine the individual association of each independent variable with CSp and CSm, respectively. Subsequently, multiple linear regression modeling was undertaken for variables exhibiting statistically significant correlations. Owing to pronounced collinearity between ChT and the associated choroidal indices, ChT was excluded from the regression models. The final models were built using a stepwise method, with probability of F-to-enter set at ≤ 0.05 and probability of F-to-remove set at ≥ 0.10, and a two-tailed *P* value < 0.05 was considered indicative of statistical significance.^23^^,^^24^

## Results

### Basic Characteristics of Patients

Table 1 delineates the baseline characteristics of all participants and elucidates the differences between the LMM and the HM groups. A total of 108 subjects were enrolled in this study, comprising 53 individuals in the LMM group (20 female subjects) and 55 individuals in the HM group (30 female subjects). The gender distribution between the two groups did not exhibit a statistically significant disparity. The mean age of the participants was 24.00 years (range = 19.00 to 29.00), with no significant age differences observed between the LMM and HM groups. The ALs were measured at 25.41 ± 0.92 mm for the LMM group and 26.93 ± 1.08 mm for the HM group. The SE refractive errors were −4.25 D (range = −4.88 to −3.50) and −8.25 D (range = −9.12 to −7.31) for the LMM and HM groups, respectively. Both AL and SE demonstrated statistically significant differences between the LMM and HM groups.

**Table 1.** Subject Demographics

Characteristics	Total (*n* = 108)	LMM (*n* = 53)	HM (*n* = 55)	Statistic	*P* Value
Age, M (Q₁, Q₃)	24.00 (19.00 to 29.00)	21.00 (18.00 to 29.00)	24.00 (21.00 to 29.00)	Z = −1.72	0.085
SE, M (Q₁, Q₃)	−6.25 (−8.25 to −4.25)	−4.25 (−4.88 to −3.50)	−8.25 (−9.12 to −7.31)	Z = −8.96	**<0.001** ^*^
AL, mean ± SD	26.18 ± 1.26	25.41 ± 0.92	26.93 ± 1.08	t = −7.84	**<0.001** ^*^
Males (%)	58 (53.70)	33 (62.26)	25 (45.45)	χ² = 3.07	0.080
Pupil diameter, mm	6.78 ± 0.05	6.87 ± 0.08	6.70 ± 0.07	t = 1.579	0.117

* *P* < 0.05 indicates statistical significance (highlighted in bold).

### Retinal and Choroidal Structural and Vascular Differences

As delineated in Table 2, the LMM group exhibited significantly greater ChT across all measured regions (0–1 mm, 1–3 mm, 3–6 mm, 0–3 mm, 1–6 mm, and 0–6 mm) compared with the HM group (*P* < 0.001 for all comparisons). No significant differences were observed in RNFT between the two groups. In the central 0 to 1 mm region of the macula, GCIPLT was significantly elevated in the HM group relative to the LMM group (*P* = 0.006), whereas no significant variations were detected at other loci. The inner nuclear layer (INL) did not differ significantly across any of the measured regions. Excluding the 0 to 1 mm range, ORT in the LMM group was markedly superior to that in the HM group (*P* < 0.01 for all comparisons). RET within the 1 to 3 mm, 3 to 6 mm, 1 to 6 mm, and 0 to 6 mm regions was significantly greater in the LMM group compared to the HM group (*P* < 0.05 for all comparisons).

**Table 2.** Regional Retinal and Choroidal Structural and Vascular Parameters

Characteristics	Total (*n* = 108)	LMM (*n* = 53)	HM (*n* = 55)	Statistic	*P* Value
RNFT					
0–1	15.61 ± 3.90	15.80 ± 3.59	15.44 ± 4.21	t = 0.48	0.633
1–3	29.88 ± 3.33	29.85 ± 3.43	29.90 ± 3.27	t = −0.08	0.939
3–6	47.25 ± 4.60	47.45 ± 4.08	47.07 ± 5.09	t = 0.43	0.670
0–6	42.51 ± 3.96	42.66 ± 3.51	42.37 ± 4.37	t = 0.37	0.711
GCIPLT					
0–1	26.88 ± 9.71	24.13 ± 7.56	29.52 ± 10.83	t = −2.99	**0.003** ^*^
1–3	83.97 ± 6.10	84.30 ± 6.53	83.65 ± 5.70	t = 0.550	0.583
3–6	55.55 ± 4.65	55.42 ± 4.50	55.67 ± 4.84	t = −0.287	0.775
0–6	61.07 ± 4.55	60.97 ± 4.54	61.16 ± 4.59	t = −0.226	0.822
INLT					
0–1	32.55 ± 4.01	32.13 ± 3.54	32.95 ± 4.42	t = −1.05	0.294
1–3	43.36 ± 2.61	43.84 ± 2.69	42.89 ± 2.47	t = 1.90	0.060
3–6	36.73 ± 1.84	36.73 ± 1.84	36.51 ± 1.99	t = 1.24	0.217
0–6	38.08 ± 1.90	38.35 ± 1.81	37.83 ± 1.96	t = 1.42	0.158
ChT					
0–1	233.54 (194.99 to 290.58)	282.27 (234.86 to 329.22)	199.85 (174.26 to 233.79)	Z = −5.801	**<** **0** **.001** ^*^
1–3	236.53 (197.18 to 289.60)	287.50 (236.82 to 335.74)	199.69 (172.92 to 236.71)	Z = −5.961	**<** **0** **.001** ^*^
3–6	239.15 (201.22 to 296.44)	284.48 (248.56 to 329.12)	210.48 (183.17 to 239.15)	Z = −5.881	**<** **0** **.001** ^*^
0–6	239.70 (199.58 to 289.17)	284.47 (245.11 to 333.14)	208.63 (181.87 to 239.70)	Z = −5.924	**<** **0** **.001** ^*^
ORT					
0–1	176.68 ± 11.52	177.22 ± 11.06	176.15 ± 12.02	t = 0.48	0.632
1–3	165.33 ± 7.89	167.52 ± 7.13	163.22 ± 8.07	t = 2.931	**0.004** ^*^
3–6	144.20 ± 7.08	146.37 ± 6.12	142.12 ± 7.37	t = 3.256	**0.002** ^*^
0–6	149.80 ± 7.08	151.93 ± 6.15	147.75 ± 7.36	t = 3.191	**0.002** ^*^
RET					
0–1	251.71 ± 20.73	249.28 ± 19.30	254.06 ± 21.93	t = −1.20	0.233
1–3	322.54 ± 13.97	325.51 ± 14.23	319.67 ± 13.22	t = 2.21	**0.029** ^*^
3–6	283.73 ± 11.88	286.18 ± 10.77	281.37 ± 12.51	t = 2.14	**0.035** ^*^
0–6	291.47 ± 11.73	293.90 ± 10.96	289.12 ± 12.06	t = 2.15	**0.034** ^*^
IRF					
0–1	0.23 (0.18 to 0.28)	0.23 (0.19 to 0.27)	0.23 (0.16 to 0.29)	Z = −0.06	0.956
1–3	5.00 (4.70 to 5.20)	5.02 (4.73 to 5.21)	4.95 (4.56 to 5.17)	Z = −1.131	0.258
3–6	16.71 (16.24 to 17.40)	17.08 (16.45 to 17.51)	16.59 (15.97 to 17.26)	Z = −2.028	**0.043** ^*^
0–6	21.95 (21.20 to 22.78)	22.05 (21.57 to 22.84)	21.66 (20.96 to 22.52)	Z = −1.862	0.063
CCF					
0–1	0.77 (0.75 to 0.77)	0.77 (0.76 to 0.78)	0.76 (0.75 to 0.77)	Z = −2.136	**0.033** ^*^
1–3	6.18 (6.10 to 6.21)	6.20 (6.13 to 6.22)	6.15 (6.06 to 6.20)	Z = −2.716	**0.007** ^*^
3–6	20.82 (20.63 to 20.96)	20.91 (20.68 to 20.99)	20.80 (20.57 to 20.89)	Z = −2.679	**0.007** ^*^
0–6	27.77 (27.50 to 27.95)	27.85 (27.56 to 27.98)	27.72 (27.38 to 27.84)	Z = −2.965	**0.003** ^*^
CVI					
0–1	0.35 ± 0.07	0.31 ± 0.06	0.38 ± 0.06	t = −6.543	**<** **0** **.001** ^*^
1–3	0.35 ± 0.05	0.32 ± 0.05	0.38 ± 0.05	t = −6.179	**<** **0** **.001** ^*^
3–6	0.34 ± 0.04	0.32 ± 0.04	0.36 ± 0.04	t = −5.212	**<** **0** **.001** ^*^
0–6	0.34 ± 0.04	0.32 ± 0.04	0.36 ± 0.04	t = −5.698	**<** **0** **.001** ^*^
CVV					
0–1	0.07 ± 0.01	0.07 ± 0.01	0.06 ± 0.01	t = 4.068	**<** **0** **.001** ^*^
1–3	0.53 ± 0.09	0.57 ± 0.08	0.48 ± 0.08	t = 5.569	**<** **0** **.001** ^*^
3–6	1.76 ± 0.33	1.93 ± 0.30	1.60 ± 0.26	t = 6.128	**<** **0** **.001** ^*^
0–6	2.35 ± 0.42	2.57 ± 0.39	2.14 ± 0.34	t = 6.057	**<** **0** **.001** ^*^

* *P* < 0.05 indicates statistical significance (highlighted in bold).

Regarding IRF, the LMM group demonstrated a significantly larger perfusion area within the 3 to 6 mm region compared with the HM group (*P* = 0.043), with no notable differences observed at other locations. CCF was significantly enhanced in the LMM group relative to the HM group across all measurement points (*P* < 0.01 for all comparisons). Conversely, CVI was significantly reduced in the LMM group compared to the HM group at all sites (*P* < 0.001 for all comparisons), whereas the CVV was significantly elevated in the LMM group relative to the HM group across all measurement points (*P* < 0.001 for all comparisons).

### Contrast Sensitivity Function

As delineated in Table 3 and the Figure, under photopic conditions, the mean CSp was significantly higher in the LMM group (1.19 ± 0.09) compared to the HM group (1.07 ± 0.11, t =6.30, *P* < 0.001). Similarly, under mesopic conditions, the median CSm in the LMM group (1.04, IQR = 0.90 to 1.11) was significantly higher than in the HM group (0.64, IQR = 0.42 to 0.81, Z = −6.16, *P* < 0.001).

**Table 3.** Contrast Sensitivity Values Under Photopic and Mesopic Conditions

Characteristics	Total (*n* = 108)	LMM (*n* = 53)	HM (*n* = 55)	Statistic	*P* Value
AULCSFp, Mean ± SD	1.13 ± 0.11	1.19 ± 0.09	1.07 ± 0.11	t = 6.30	**<0.001** ^*^
AULCSFm, M (Q₁, Q₃)	0.83 (0.59 to 1.05)	1.04 (0.90 to 1.11)	0.64 (0.42 to 0.81)	Z = −6.16	**<0.001** ^*^

* *P* < 0.05 indicates statistical significance (highlighted in bold).

**Figure.**
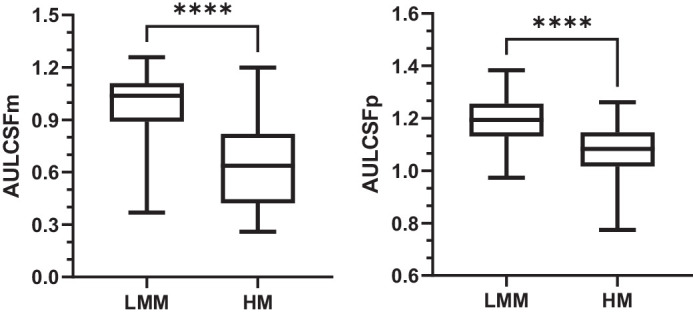
Contrast sensitivity of subjects in the LMM and HM groups under photopic and mesopic conditions. ****P < 0.001.

### Relationship Between Contrast Sensitivity Function and Retinal and Choroidal Structural and Vascular Parameters Within the 0 to 6 mm macular range

Univariate linear regression analysis (Table 4) revealed that within the 0 to 6 mm macular range, sex, age, AL, ChT, CVI, CVV, and IRF were all significantly associated with both photopic contrast sensitivity (CSp) and mesopic contrast sensitivity (CSm; all *P* < 0.05). Specifically, male sex, greater ChT, larger CVV, and higher IRF were positively correlated with both CSp and CSm, whereas increasing age, longer AL, and higher CVI were negatively correlated with both.

**Table 4.** Univariate Linear Regression Analysis of Photopic Contrast Sensitivity (CSp) and Mesopic Contrast Sensitivity (CSm) With Various Factors

	CSp			CSm		
	Std. Beta	Adjusted *R*²	*P* Value	Std. Beta	Adjusted *R*²	*P* Value
Sex	0.297	0.080	**0.002** ^*^	0.256	0.056	**0.008** ^*^
Age	−0.271	0.065	**0.005** ^*^	−0.290	0.076	**0.002** ^*^
AL	−0.257	0.057	**0.007** ^*^	−0.297	0.079	**0.002** ^*^
Pupil diameter	−0.0530	−0.007	0.584	0.032	−0.008	0.742
RNFT	−0.030	−0.009	0.755	−0.068	−0.005	0.482
GCIPLT	0.056	−0.006	0.562	0.180	0.023	0.062
INLT	0.080	−0.003	0.413	0.090	−0.001	0.354
ChT	0.332	0.102	**<0.001** ^*^	0.393	0.147	**<0.001** ^*^
ORT	0.105	0.002	0.279	0.259	0.058	**0.007** ^*^
IRF	0.198	0.030	**0.040** ^*^	0.292	0.077	**0.002** ^*^
CCF	0.185	0.025	0.055	0.189	0.022	0.066
CVI	−0.320	0.094	**0.001** ^*^	−0.374	0.131	**<0.001** ^*^
CVV	0.281	0.070	**0.003** ^*^	0.336	0.104	**<0.001** ^*^

Std. Beta: Standardized Beta Coefficient; Adjusted R²: Adjusted R-squared; P: P-value.

* *P* < 0.05 indicates statistical significance (highlighted in bold).

All listed fundus parameters are measurements within the 0 to 6 mm macular range.

Notably, for all the aforementioned statistically significant parameters, their associations with CSm generally exhibited larger absolute standardized beta coefficients and higher adjusted R² values compared with their associations with CSp. Furthermore, ORT demonstrated a significant positive correlation only with CSm (Std. Beta = 0.259, *P* = 0.007), whereas its association with CSp did not reach statistical significance. In contrast, RNFLT, GCIPLT, INLT, CCF, and pupil diameter showed no significant association with either CSp or CSm (all *P* > 0.05). The detailed results of the univariate linear regression analyses quantifying the effect of each diopter of spherical equivalent on CS and choroidal parameters are provided in Supplementary Table S1.

As delineated in Tables 5 and 6, multiple linear regression analyses revealed the associations between CS and various chorioretinal parameters.

**Table 5.** Parameters Exhibiting Statistical Significance in the Multiple Regression Analysis Under Mesopic Conditions

	Unstandardized Coefficient		Standardized Coefficient			
Parameters	B	Standard Error	Beta	95% CI		*P* Value
0–1 mm^†^						
Age	−0.008	0.004	−0.187	−0.017	0.000	0.060
AL	−0.068	0.024	−0.306	−0.115	−0.021	**0.005** ^*^
Sex	0.133	0.059	0.237	0.016	0.250	**0.026** ^*^
CVI	−0.714	0.415	−0.175	−1.538	0.109	0.088
1–3 mm^‡^						
Age	−0.012	0.004	−0.258	−0.019	−0.004	**0.004** ^*^
CVI	−1.819	0.446	−0.352	−2.704	−0.934	**<0.001** ^*^
IRF	0.110	0.051	0.187	0.010	0.211	**0.032** ^*^
3–6 mm^§^						
Age	−0.016	0.004	−0.350	−0.023	−0.008	**<0.001** ^*^
CVI	−1.946	0.571	−0.283	−3.077	−0.814	**0.001** ^*^
IRF	0.079	0.020	0.336	0.040	0.119	**<0.001** ^*^

† 0–1 mm Model: R² = 0.270, Adjusted R² = 0.241. *P* < 0.001.

‡ 1–3 mm Model: R² = 0.259, Adjusted R² = 0.238. *P* < 0.001.

§ 3–6 mm Model: R² = 0.305, Adjusted R² = 0.285. *P* < 0.001.

**Table 6.** Parameters Exhibiting Statistical Significance in the Multiple Regression Analysis Under Photopic Conditions

	Unstandardized Coefficient		Standardized Coefficient			
Parameters	B	Standard Error	Beta	95% CI		*P* Value
0–1 mm^†^						
Sex	0.095	0.021	0.414	0.054	0.136	**<0.001** ^*^
AL	−0.035	0.008	−0.383	−0.051	−0.019	**<0.001** ^*^
1–3 mm^‡^						
Sex	0.083	0.022	0.360	0.039	0.126	**<0.001** ^*^
CVI	−0.367	0.216	−0.174	−0.794	0.061	0.092
AL	−0.026	0.010	−0.286	−0.045	−0.007	**0.008** ^*^
3–6 mm^§^						
Sex	0.095	0.021	0.414	0.054	0.136	**<0.001** ^*^
AL	−0.035	0.008	−0.383	−0.051	−0.019	**<0.001** ^*^

† 0–1 mm Model: R² = 0.221, Adjusted R² = 0.207. *P* < 0.001.

‡ 1–3 mm Model: R² = 0.242, Adjusted R² = 0.221. *P* < 0.001.

§ 3–6 mm Model: R² = 0.221, Adjusted R² = 0.207. *P* < 0.001.

* *P* < 0.05 indicates statistical significance (highlighted in bold).

For CSm, in the central fovea (0–1 mm), the model explained 24.1% of the variance (adjusted *R*² = 0.241), with AL (Std. β = −0.306) and sex (Std. β = 0.237) as significant independent predictors (*P* < 0.05). The CVI (*P* = 0.088) and age (*P* = 0.060) were also retained with a borderline association. In the parafoveal region (1–3 mm), the model (adjusted *R*² = 0.238) identified CVI (Std. β = −0.352), age (Std. β = −0.258), and IRF (Std. β = 0.187) as significant predictors (*P* < 0.05). Similarly, the perifoveal model (3–6 mm) showed the highest explanatory power (adjusted *R*² = 0.285), with age (Std. β = −0.350), IRF (Std. β = 0.336), and CVI (Std. β = −0.283) remaining as significant independent predictors (*P* < 0.001).

For CSp, in both the 0 to 1 mm and 3 to 6 mm ranges, the models were identical, explaining 20.7% of the variance (adjusted *R*² = 0.207) with sex (Std. β = 0.414) and AL (Std. β = −0.383) as the significant predictors (*P* < 0.001). The model for the 1 to 3 mm range explained 22.1% of the variance (adjusted *R*² = 0.221); whereas AL (Std. β = −0.286) and sex (Std. β = 0.360) were significant predictors (*P* < 0.001), CVI (*P* = 0.092) was also retained with only a borderline trend.

## Discussion

This study found that, compared to the LMM group, significant chorioretinal structural and vascular alterations are present in the high myopia group, and that these alterations are closely associated with a decline in visual function, as measured by CS. These findings are consistent with previous studies, which have indicated that structural and vascular changes driven by axial elongation, including choroidal thinning,^20^^,^^25^ alterations in retinal thickness,^26^^,^^27^ and diminished blood perfusion,^28^ are primary contributing factors to myopia-related decreases in visual function.^9^^,^^29^^,^^30^ In addition, this study found that the strength of this structure-function association differs significantly under various lighting conditions, with mesopic vision exhibiting a higher sensitivity to these pathophysiological changes.

This study demonstrated that choroidal remodeling in high myopia is closely associated with visual function decline. The results showed that among various parameters, CVI, a quantitative measure of the vascular-to-stromal ratio within the choroid, was significantly and negatively associated with both photopic and mesopic contrast sensitivity. This highlights the functional impact of these deep structural changes and establishes CVI as a noteworthy parameter in the context of myopia-related visual impairment.

The behavior of the CVI in myopia is a subject of debate. Whereas some studies report a decrease in CVI, attributing it to overall choroidal vascular atrophy,^31^ others have observed an increase. This latter finding is often explained by a disproportionate loss of choroidal stroma relative to the vascular components, which paradoxically elevates the CVI ratio.^32^^–^^34^ Recently, a more nuanced, nonlinear relationship has been proposed: CVI may first increase as stromal atrophy outpaces vascular atrophy in the early stages of myopia,^35^ and then decrease in later stages, such as pathological myopia, when vascular loss becomes dominant.^20^ The finding that the CVI in the HM group was significantly higher than in the LMM group is consistent with the first phase of this nonlinear model, as the sample did not include subjects with pathological myopic maculopathy. Crucially, the multiple regression analysis identified CVI as a significant independent predictor for mesopic CS, whereas its predictive role for photopic vision was only borderline. This suggests that the altered vascular-to-stromal ratio reflected by CVI is not merely an anatomic observation but is functionally significant. Therefore, in patients with non-pathological myopia, CVI represents a noteworthy parameter associated with functional changes, and it warrants further investigation as a potential biomarker for assessing visual performance.

One of the core findings of this study is that mesopic visual function exhibits a more intimate and extensive association with chorioretinal structural and vascular alterations in myopia than its photopic counterpart. The univariate regression analysis provided initial evidence for this trend: the associations with CSm were generally stronger, reflected by larger standardized beta coefficients and higher adjusted R-squared values. Furthermore, in the multivariate regression models, CSm was independently predicted by a greater number of chorioretinal parameters. Most notably, the IRF was identified as an independent predictor for CSm in the parafoveal and perifoveal regions, whereas it lacked any such independent association in the models for CSp. Collectively, these findings provide strong evidence that mesopic vision serves as a more sensitive window for assessing the functional impairment resulting from the pathophysiological chorioretinal alterations in myopia.

Previous studies have indicated that under low-light conditions, the retina’s demand for oxygen and energy significantly increases, primarily due to the high metabolic activity of rod photoreceptors.^17^ During dark adaptation, rod cells require substantial amounts of adenosine triphosphate (ATP) for the resynthesis of rhodopsin, which is a critical step in maintaining visual sensitivity.^36^^,^^37^ In dark environments, rod cells remain in a depolarized state, a process that demands significant energy to maintain membrane potential stability,^38^ Additionally, the neurotransmitter glutamate is continuously and actively released from the photoreceptors’ ribbon synapses, which is essential for sustaining signal transmission to downstream bipolar cells,^39^ Therefore, rod cells are in a state of high metabolic activity in dark environments to meet their energy and oxygen consumption needs, placing greater demands on the blood supply to the choroid and retina.^40^^,^^41^

However, in patients with high myopia, degenerative alterations in fundus structure lead to a significant decline in choroidal oxygenation capacity.^42^^,^^43^ The reduction in blood flow within the choroid and retina fails to meet the high metabolic demands of retinal outer-layer neurons, particularly under dark or low-light conditions, exacerbating the supply-demand imbalance.^38^^,^^44^ Established research confirms that rods, which are critical for mesopic vision, predominate outside the cone-dense fovea.^45^^,^^46^ This anatomic layout is closely mirrored by the spatial dependency observed in this study’s findings: the correlation between CSm and vascular parameters was confined specifically to these rod-dominant parafoveal and perifoveal regions. This study is consistent with this theory and suggests that patients with high myopia are more prone to oxygen insufficiency under low-light conditions. Consequently, the decline in CS under mesopic conditions more accurately reflects the extent of visual function and fundus structural impairment. Therefore, conducting CS tests in dark environments can serve as a valuable and sensitive indicator for assessing the degree of visual function and fundus structural impairment in patients with myopia, holding potential clinical relevance for diagnosis and treatment.

The correlation identified between CSm and chorioretinal structural parameters carries significant clinical implications. This study suggests that CSm can act as a sensitive functional correlate of fundus health, forming the basis for a reciprocal clinical alert system. Specifically, an unexplained decline in CSm should prompt detailed structural evaluation, whereas observed structural alterations should trigger a functional assessment under mesopic conditions. Although this approach enhances clinical vigilance, the cross-sectional design of the present study limits the interpretation to association, precluding conclusions on causation or prediction. Therefore, establishing CSm as a predictive biomarker for early warning necessitates future prospective, longitudinal studies.

Nevertheless, this study has certain limitations. First, the investigation was structured to compare low-to-moderate and high myopia to characterize the alterations that occur across the myopic spectrum. Therefore, an emmetropic control group was not included, which means the conclusions are confined to the relative differences between these severities of myopia, rather than defining the absolute functional impairment relative to a non-myopic state. Consequently, the LMM group serves only as an internal reference, not a physiological norm. Second, whereas the sample size provided sufficient statistical power for this analyses, the cohort did not include individuals with emmetropia or pathological myopia. Future research should therefore aim to include a larger and more diverse cohort to more comprehensively understand the impact of varying degrees of the condition and to enhance the generalizability of the findings. Finally, because glare can significantly impact visual function and its assessment, future research will investigate how the relationship between CS and fundus structure is altered in such environments.

In conclusion, this study confirmed that significant chorioretinal structural and hemodynamic alterations in highly myopic eyes are closely associated with a decline in visual function, with this structure-function association being significantly more pronounced and extensive under mesopic than photopic conditions. This underscores the importance of the mesopic visual system as a more sensitive indicator for assessing the underlying chorioretinal pathophysiological changes in myopia. Therefore, evaluating visual performance under low-light conditions may provide crucial insights for the comprehensive assessment and management of visual impairment in patients with high myopia.

## Supplementary Material

Supplement 1
