# Supplementary material for: The Relationship of Photopic and Mesopic Contrast Sensitivity to Retinal–Choroidal Structural Characteristics in Low-to-Moderate and High Myopia
Source: Transl Vis Sci Technol. 2025 Dec 2;14(12):6. doi: 10.1167/tvst.14.12.6 (PMC12697708; doi:10.1167/tvst.14.12.6)
Supplement: Supplement 1 [file tvst-14-12-6_s001.pdf]

Table S1. Univariate Linear Regression Analyses for the Effect of Spherical Equivalent (SE) on Contrast Sensitivity and Choroidal Parameters.

		CSp	CSm	ChT	CCF	CVV	CVI
SE	B	0.022	0.066	16.507	0.034	0.085	-0.009
	Adjusted R^2	0.201	0.293	0.288	0.041	0.214	0.237
	P	<0.001*	<0.001*	<0.001*	0.020*	<0.001*	<0.001*

B: Unstandardized coefficient.

Abbreviations: SE, Spherical Equivalent; CSp, Photopic Contrast Sensitivity; CSm, Mesopic Contrast Sensitivity; ChT, Choroidal Thickness; CCF, Choriocapillaris Flow; CVV, Choroidal Vascular Volume; CVI, Choroidal Vascularity Index.

*: $p < 0.05$.
